# Series 2: Development of a Multiplex Amplicon Next Generation Sequencing Assay for Rapid Assessment of Resistance-Associated Mutations in *M. tuberculosis* Clinical Cases

**DOI:** 10.3390/tropicalmed10070194

**Published:** 2025-07-10

**Authors:** Adriana Cabrera, Tracy Lee, Kathleen Kolehmainen, Trevor Hird, Danielle Jorgensen, Calvin Ka-Fung Lo, Hasan Hamze, Alan O’Dwyer, Dan Fornika, Rupinder Kaur KhunKhun, Mabel Rodrigues, Natalie Prystajecky, John Tyson, James E. A. Zlosnik, Inna Sekirov

**Affiliations:** 1 British Columbia Centre for Disease Control Public Health Laboratory, Vancouver, BC V5Z 4R4, Canada; adriana.cabreradelgado@phsa.ca (A.C.); tracy.lee@bccdc.ca (T.L.); kathleen.kolehmainen@bccdc.ca (K.K.); trevor.hird@bccdc.ca (T.H.); danielle.jorgensen@bccdc.ca (D.J.); alan.odwyer@phsa.ca (A.O.); dan.fornika@bccdc.ca (D.F.); rupinder.khunkhun@bccdc.ca (R.K.K.); mabel.rodrigues@bccdc.ca (M.R.); natalie.prystajecky@bccdc.ca (N.P.); john.tyson@bccdc.ca (J.T.); jzlosnik@bccdc.ca (J.E.A.Z.); 2 Department of Pathology and Laboratory Medicine, Faculty of Medicine, University of British Columbia, Vancouver, BC V5Z 3N9, Canada; calvinlo66@alumni.ubc.ca (C.K.-F.L.); h.hamze@alumni.ubc.ca (H.H.)

**Keywords:** tuberculosis, antibiotic resistance, next generation sequencing, molecular

## Abstract

Treatment of *Mycobacterium tuberculosis* requires multi-drug regimens, and resistance to any individual antibiotic can compromise outcomes. For slow-growing organisms like *M. tuberculosis*, rapid detection of resistance-conferring mutations enables timely initiation of effective therapy. Conversely, confirming wild-type status in resistance-associated genes supports confidence in standard regimens. We developed an amplicon-based next generation sequencing (amplicon tNGS) assay on the Illumina platform targeting eight genes linked to resistance to isoniazid, rifampin, ethambutol, pyrazinamide, and fluoroquinolones. Sequencing results were analyzed using a custom bioinformatics pipeline. Forty-seven samples were used for assay development, and 37 additional samples underwent post-implementation clinical validation. Compared to whole genome sequencing (WGS), amplicon tNGS demonstrated 97.7% sensitivity, 98.9% specificity, and 98.7% overall accuracy for variant detection in targeted regions. Resistance prediction showed 79.3% concordance with WGS; discrepancies were primarily due to mutations outside of target regions. Among post-implementation samples, 27/37 passed quality metrics for all targets, with 95.7% concordance between amplicon tNGS results and final susceptibility results. This assay is now in use in our laboratory and offers significantly faster turnaround than both WGS and phenotypic methods on cultured isolates, enabling more rapid, informed treatment decisions for tuberculosis patients.

## 1. Introduction

*Mycobacterium tuberculosis* (MTB) is a pathogen that causes tuberculosis (TB) infection and disease; it has been devastating human populations for millennia [1], and despite longstanding and ongoing efforts by the medical and scientific communities, elimination of TB is yet to be achieved. The WHO has set ambitious targets towards TB eradication [2]; however, significant additional work is needed to achieve them, especially given the setbacks to TB elimination experienced during the COVID-19 pandemic [3].

Drug resistance of MTB remains a global concern; although some areas of the world have seen downward trends in incidence of drug-resistant MTB (DR-MTB), cases remain worldwide and have been climbing in some countries [4]. The WHO recommends universal drug susceptibility testing for all cases of TB disease, with genotypic testing being an acceptable testing modality [2]. Rapid detection of MTB resistance markers is key to starting patients on effective TB treatment quickly, facilitating treatment and reducing unnecessary toxicities. Many genetic targets for *M. tuberculosis* drug resistance have been successfully validated [5], and the WHO has published an updated catalogue of mutations in 2023 [6].

In addition to effective treatment initiation considerations, rapid evaluation of drug resistance/susceptibility patterns also plays a key role for timely discontinuation of patient isolation precautions. Canadian de-isolation guidelines changed in 2022, with current Canadian TB Standards [7] recommending discontinuation of airborne precautions for drug-susceptible pulmonary TB after 2–4 weeks of effective therapy with demonstrated clinical improvement (duration dependent on original AFB smear-positivity), and in cases of rifampin-resistant TB, after at least 4 weeks of effective therapy, when second line drug susceptibilities are available and at least three consecutive sputa have become AFB smear-negative for baseline positive patients. Of note, genotypic predictions of antibiotic susceptibility were considered adequate for this kind of decision making. Even more drastically, in April 2024, the National Tuberculosis Coalition of America recommended that most patients in community settings with TB can be de-isolated after 5 days of effective treatment, again with molecular testing being considered suitable for determination of treatment effectiveness [8]. Given these significant changes in de-isolation recommendations, which stand to significantly benefit the patients’ psychosocial well-being, as well as decrease isolation burdens on the healthcare system, it is imperative for clinical laboratories to be able to not only quickly detect potentially resistant cases, but also rapidly assess for genotypically predicted susceptibility to first line agents.

The WHO provides consolidated guidelines on tuberculosis, including for rapid diagnostic options [9]. This most recent version of guidelines, published in 2024, discusses both rapid molecular detection options and targeted next generation sequencing (tNGS)-based options. The selection of target drugs for rapid drug resistance prediction is variable for WHO-endorsed tests, with the majority including rifampin and variable inclusion for isoniazid and second line drug targets. For tNGS commercial options, the WHO recommends inclusion of rifampin, isoniazid, quinolone and bedaquiline as minimal requirement, with several caveats (not fully elucidated molecular basis of resistance, cost-effectiveness) included for bedaquiline recommendation [10]. A thorough review and meta-analysis was recently published on the available commercial and laboratory developed tNGS assays for diagnosis of drug-resistant tuberculosis [11], with reported performance characteristics affected in part by selection of enrolled samples (primary vs. cultured isolates and all-comers vs. only patients with drug-resistant tuberculosis). However, the overall performance was high among all included studies, with >90% sensitivity and >97% specificity overall, with some unevenness in performance for individual drug targets. For published laboratory developed amplicon tNGS assays, there is variability between included drug targets, with some centers choosing to focus on confirmation of MDR-TB profile [12], and others including additional targets, such as quinolones, ethambutol, linezolid, and injectables, on their panel [13,14,15,16].

Given the landscape of current diagnostic trends, our laboratory worked to develop a cost-effective amplicon tNGS assay for rapid assessment of TB AMR profiles. Our choice of targets was based on our local TB resistance landscape, availability of follow-up testing (both genotypic and phenotypic), and cost effectiveness and workflow integration considerations. In our jurisdiction, as well as in many developed countries in general, isoniazid monoresistance is the most commonly encountered resistance pattern [17]. Consequently, we chose to include multiple isoniazid resistance-associated genes on our assay, including those with already described high confidence resistance markers (*inh*A/*fab*G1, *kat*G), as well as some that do not currently have a high confidence grading from the WHO, but have been described in the literature in isoniazid-resistant isolates (*ndh*, *ahp*C). We included the rifampin resistance-determining region for rapid MDR-TB profile detection, *emb*B target for ethambutol (as it accounted for >95% of ethambutol resistance-associated mutations in our jurisdiction identified over nearly 2 decades, and 100% of those with phenotypic resistance concordance), *pnc*A target for pyrazinamide (to complete assessment of first line regimen drugs), and *gyr*A target for quinolones (as it accounted for 87.5% of quinolone resistance-associated mutations in our jurisdiction identified over nearly 2 decades and 100% of those with phenotypic resistance concordance), as quinolones are the most common drug substituted for isoniazid for treatment of isoniazid monoresistant TB cases. Here, we describe our assay design, its performance characteristics, and laboratory implementation, and highlight its clinical utility.

## 2. Materials and Methods

### 2.1. Samples

There were 47 *Mycobacterium tuberculosis* complex (MTBC) samples assessed for amplicon-based antimicrobial resistance (AMR) detection from a previously established validation panel. These were previously identified as MTBC through *hsp65* sequencing speciation (see Lee et al. “The use of *hsp65* and *erm41* targeted amplicon sequencing in non-tuberculous mycobacteria diagnostic workflow” Series 1 Paper, Supplementary Table S1). Of these 47 MTB samples, 16 were provided as isolates from Canada’s National Microbiology Laboratory (NML), 14 were culture samples, and 17 were primary patient samples (Supplementary Table S1). The primary patient samples included sputum (11), ascitic fluid (1), neck abscess (1), bronchial washings (2), pleural fluid (1), and cecum tissue (1) (Supplementary Table S1). The MTBC control strain H37Rv (sensitive) was used to assess the limit of detection (LOD) and precision for amplicon-based AMR detection. An additional 37 MTBC samples were assessed post-implementation, including 29 primary patient or direct samples and 8 culture samples. Sample types for primary patient samples include sputum (16), tissue (3), gastric aspirate (1), CAP samples (2), scrolls (2), abscess (1), bronchiolar washes (2), bronchoalveolar lavage (1), and upper tract urothelial carcinoma (1) (Supplementary Table S5). These samples were collected between October 2023 and June 2025.

### 2.2. Assay Parameters

Sample extraction, *mpt*64 qPCR assay, target amplification, library preparation, and sequencing methods are identical to those used in the initial *hsp65*-based speciation validation study (see Lee et al. “The use of *hsp65* and *erm41* targeted amplicon sequencing in non-tuberculous mycobacteria diagnostic workflow” Series 1 Paper). Primers used for AMR-specific amplicon targeting are shown in Table 1.

### 2.3. Bioinformatics

Using the same bioinformatics pipeline as described in Lee et al., “The use of *hsp*65 and *erm*41 targeted amplicon sequencing in non-tuberculous mycobacteria diagnostic workflow” Series 1 Paper, (https://github.com/BCCDC-PHL/auto-tb-amr-amplicon; accessed on 28 April 2025), samples identified as members of the MTBC complex (*M. tuberculosis, M. bovis* or *M. caprae*) were processed by tbProfiler [v.6.2.0] for molecular AMR prediction (https://github.com/jodyphelan/TBProfiler; accessed on 28 April 2025). The NGS pipeline evaluates 8 gene targets that can harbor common resistance mutations to rifampicin, isoniazid, pyrazinamide, ethambutol, and fluoroquinolones: *rpo*B, *kat*G, *inh*A/*fab*G1, *ndh*, *ahp*C, *pnc*A, *emb*B, and *gyr*A (Table 1). Quality control (QC) checks were implemented in the NGS pipeline to evaluate the targets’ completeness before AMR detection by tbProfiler. For the analysis, the definition of “PASS” criterion is an amplicon with a coverage of ≥90% relative to reference for all 8 target amplicons. If at least 1 out of 8 amplicons had an amplicon coverage of <90%, the sample was flagged as “REVIEW/REPEAT”. If the *pnc*A amplicon had a p.His57Asp mutation detected, the NGS pipeline reports this sample as “presumptive *M. bovis*/*M. bovis* BCG”. All analyses were completed using version 0.2.2 of this pipeline.

### 2.4. AMR Reference Method

For AMR detection, mutations identified by the NGS pipeline were compared to those identified through whole genome sequencing (WGS). For primary patient samples, amplicon AMR mutations were compared to WGS results obtained on cultured MTBC isolates from the same sample. The extraction and library preparation for WGS is identical to that for NGS. AMR detection from WGS was completed using tbProfiler as part of the BCCDC-PHL/tbprofiler-nf pipeline (https://github.com/BCCDC-PHL/tbprofiler-nf; accessed on 28 April 2025), a nextflow-based wrapper for the tbProfiler tool.

### 2.5. Phenotypic Susceptibility Testing

Susceptibility testing of NML isolates was carried out by NML using the BD BACTEC^TM^ MGIT^TM^ 560 or 960 Mycobacteria detection system (BD) and testing at the following concentrations: 5 μg/mL ethambutol, 100 μg/mL pyrazinamide, 2 μg/mL moxifloxacin, and 2 μg/mL ofloxacin. Susceptibility testing of all other samples was completed in the BCCDC public health laboratories using the BD BACTEC^TM^ MGIT^TM^ 960 Mycobacteria detection system (BD) on pure MTBC cultures at a 0.5 McFarland standard. Recommended critical breakpoint concentrations were used for all antimicrobials: 1 μg/mL rifampin (Cat#: 245123, Becton, Dickinson and Company, Franklin Lakes, NJ, USA), 0.1 μg/mL isoniazid (Cat#: 245123, Becton, Dickinson and Company, Franklin Lakes, NJ, USA), 100 μg/mL pyrazinamide (Cat#: 245128, Becton, Dickinson and Company, Franklin Lakes, NJ, USA), 5 μg/mL ethambutol (Cat#: 245123, Becton, Dickinson and Company, Franklin Lakes, NJ, USA), and 2 μg/mL ofloxacin (Cat#: 087570-1G, Millipore Sigma, Oakville, ON, Canada).

### 2.6. Validation Parameters

Amplicon-based AMR detection was assessed for accuracy and precision. To evaluate the accuracy, MTBC samples were assessed for (1) the number of mutations detected and (2) the accuracy of the mutation call. Only the AMR targets (*rpo*B, *kat*G, *inh*A/*fab*G1, *ndh*, *ahp*C, *pnc*A, *emb*B, and *gyr*A) and their associated antibiotics were included in the validation. Definitions for accuracy, sensitivity, and specificity assessment of variant detection using a 2 × 2 contingency table were as follows: true positive = variant in NGS also present in WGS; true negative = variant absent in NGS also absent in WGS; false positive = variant present in NGS but not in WGS; and false negative = variant absent in NGS but present in WGS. A concordance calculation was also completed for predicted drug resistance type through NGS and WGS, and for NGS predicted resistance/sensitivity to select antibiotics when compared to results from susceptibility testing. Precision and LOD for sequencing of AMR amplicons were evaluated using H37Rv culture serially diluted 1:10 from 10^−2^ to 10^−7^. Briefly, pure H37Rv culture was harvested from solid Lowenstein Jennings slants into 500 μL of 0.5× TBE buffer and heat-killed at 95 °C for 10 min. Serial dilutions were made from this culture lysate in 1× IDTE buffer and sequenced in triplicate over 1–3 runs.

### 2.7. Statistics

Two-sided 95% confidence intervals were calculated for accuracy, analytical specificity, and analytical sensitivity using the Wilson score method as outlined in EP12 and Westgard QC [26].

### 2.8. Clinical Review of Turnaround Times

To compare the prospective clinical impact of broader availability of direct molecular resistance testing on primary patient samples, the turnaround times (TAT) for phenotypic susceptibility results of smear-positive cases (those most amenable to direct molecular resistance testing) diagnosed in BC in 2023 were reviewed for comparison with 4 days TAT (from AFB smear results or an AFB smear-positive sample arrival in the laboratory) of amplicon MDR NGS test. Laboratory data were extracted from the BCCDC PHL Laboratory Information System. Results of 343 TB cases (*n* = 234 pulmonary TB; *n* = 82 extrapulmonary TB (EPTB); *n* = 27 pulmonary and extrapulmonary TB) were included for review. Phenotypic susceptibility results TATs were assessed relative to the day of sample collection and to the day of AFB smear results availability (i.e., the day when the sample was processed in a laboratory).

## 3. Results

### 3.1. Analytical Accuracy, Sensitivity, and Specificity of AMR Variant Detection in Validation Samples

The accuracy of tbProfiler mutation calling for amplicon-based AMR detection was assessed using 29 of 47 MTBC samples for which the AMR detection passed the QC criteria and for which WGS results were available (Supplementary Tables S2 and S3). There were 42 true positive variants, 187 true negative variants, 2 false positive variants, and 1 false negative variant (Table 2) giving a calculated accuracy of 98.7% (95% CI: 96.3–99.6%), a calculated sensitivity of 97.7% (95% CI: 87.9–99.6%), and a calculated specificity of 98.9% (95% CI: 96.2–99.7%). The three discrepant samples were 20s304, which had a *pnc*A p.P54S in NGS but not WGS; PS3, which had an *emb*B p.M306V variant present in NGS but not WGS; and PS4, which had a *kat*G p.S315T variant present in WGS but not NGS (Table 3).

### 3.2. Concordance of Drug Resistance Type Between NGS and WGS

Based on variants detected through NGS and WGS, a prediction of DR type was made by the pipeline. The DR type matched for 23/29 samples giving a calculated concordance of 79.3%. The discrepant samples were 23s137, PS1, PS4, PS7, PS9, and SPEC-34 (Table 3). Sample 22s137 was called “RR-TB” by NGS but “MDR-TB” by WGS due to the presence of an *inh*A mutation (p.I194T) outside the amplicon target range. The other discrepant samples were all called “Sensitive” by NGS but “HR-TB” (PS1, 4, 7, and 9) or “Other” (SPEC-34) by WGS. Three of these samples (PS1, 7, and 9) similarly had an *inh*A mutation (c.-154G > A) outside the amplicon target region, while one sample (PS4) had a discrepancy in mutation detection between NGS and WGS (*kat*G p.S315T). The final sample (SPEC-34) did not have any discrepant variants in the AMR amplicons included in NGS but did have a *gyr*B variant (p.Glu501Asp) detected by WGS that indicated resistance to fluoroquinolones. The *gyr*B is not part of the AMR target amplicons generated in this assay.

### 3.3. Limit of Detection

The LOD of amplicon detection was *mpt*64 Ct 35.81 (10^−5^ dilution) as all 8/8 amplicons in H37Rv were detected at this Ct with full length amplicon (Table 4 and Table S4). Note that 1/9 10^−5^ replicates had a single SNP (p.Asp136Tyr) in *pnc*A representing a low confidence mutation (Supplementary Table S4). This SNP was identified as a degenerate base (Supplementary Figure S1) and is likely a PCR artifact which can randomly occur, especially with low copy template samples.

### 3.4. Precision

H37Rv dilutions from 10^−1^ down to 10^−5^ had 100% median coverage across all eight AMR amplicons for all replicates (Table 4 and Table S4). Additionally, H37Rv was accurately predicted as “Sensitive” for culture dilutions of 10^−1^ to 10^−4^, with no mutations detected in any replicates (Supplementary Table S4). At 10^−5^, s1/9 H37Rv replicates typed as “Other”. The detected mutation, *pnc*A p.Asp136Tyr, is likely a PCR artifact as previously described in LOD.

### 3.5. Phenotypic vs. Genotypic Susceptibility Testing Results

Susceptibility testing was carried out by NML on NML isolates and by BCCDC PHL for all other validation samples. There were 12 NML isolates that had phenotypic susceptibility testing results and passed NGS amplicon QC criteria. Antibiotics tested by NML included ethambutol, pyrazinamide, ofloxacin, and moxifloxacin. There was 100% concordance between phenotypic susceptibility results for each antibiotic and resistance mutations called by tbProfiler using the amplicon-based NGS method (Table 5). There were an additional 29 samples for which susceptibility testing was completed by BCCDC PHL and samples passed NGS amplicon QC criteria. Antibiotics tested included rifampin, isoniazid, ethambutol, pyrazinamide, and ofloxacin. Note that pyrazinamide testing was only completed for 14 samples. The calculated concordance was 90% for rifampicin (three discrepant), 83% for isoniazid (five discrepant), 90% for ethambutol (three discrepant), 93% for pyrazinamide (one discrepant), and 100% for ofloxacin (zero discrepant) (Table 6). Note that five of these samples (23s137, PS1, PS4, PS7, and PS9) also had DR prediction discrepancies between NGS and WGS due to the presence of an *inh*A variant outside the amplicon target range or a discrepant *kat*G variant (Table 3). An additional sample (22s317) also had an *emb*B variant detected by WGS that was outside the amplicon target range, which could account for the ethambutol resistance detected by susceptibility testing (Table 3). Pyrazinamide resistance was also detected for 18s423 although no *pnc*A mutation was present by NGS or WGS (Supplementary Tables S2 and S3). The remaining five samples (18s262, 18s514, 19s078, 20s291, and 22s305) were all sensitive to rifampicin or ethambutol despite the presence of putative resistance mutations (in amplicons *rpo*B and *emb*B, respectively) (Table 6).

### 3.6. Post-Implementation Clinical Samples

There were an additional 37 samples collected post-implementation that were identified as MTBC through *hsp65* speciation. Of these, 27 passed NGS QC metrics (8/8 amplicons with coverage ≥ 90%). This included 21/29 direct samples and 6/8 culture samples (Table 7). Low (to no) coverage of the *gyr*A amplicon occurred in 9/10 samples that failed QC. Additional failed amplicons and sample information can be found in Supplementary Table S5. Phenotypic AST was available for 23 samples that passed QC and was concordant with DR variants identified through the pipeline in 22/23 samples (95.7%). For CS_9, although the DR type called by the pipeline (MDR-TB; classified by resistance to at least isoniazid and rifampicin) was concordant with the MDR phenotypic AST resistance profile, ethambutol resistance was not predicted by the pipeline despite the sample being ethambutol-resistant. It is possible that the variant conferring ethambutol resistance is outside the *emb*B amplicon target range. CS_23 was a discordant sample as the DR type was sensitive with no DR variants detected, while the phenotypic AST was repeatedly moxifloxacin-resistant. Again, this could point to a resistance mutation being outside the amplicon target range or in a different target, such as *gyr*B. These results underscore the need for concurrent WGS and phenotypic AST on growing organisms to confirm any potential drug resistance phenotypes identified through sequencing. However, the high rate of concordance found between amplicon NGS-based predictions and final phenotypic AST (95.7%) does support use of the DR amplicon panel and tbProfiler pipeline for rapid drug resistance prediction.

### 3.7. Detection of M. bovis/M. bovis BCG-Specific pncA Mutation

Sequencing of pncA allows for “presumptive *M. bovis*/*M. bovis* BCG” assignment based on the presence of pHis57Asp variant in this amplicon. There were three validation samples and six clinical samples in which this variant was detected (Table 8). The predicted *M. bovis/M. bovis* BCG presence matched the reference ID for all three validation samples.

### 3.8. Results of Clinical Review of Turnaround Times

The AFB smear positivity among reviewed cases was as follows: pulmonary TB 125 AFB-pos cases (53.4%), EPTB 22 AFB-pos cases (26.8%), both pulmonary and EPTB 18 AFB-pos cases (66.7%). The patients could be further classified based on their *mpt*64 Ct value, for more granular delineation of those samples that would have been most successfully tested using amplicon MDR NGS test (see Table 9).

Of the AFB-Pos samples with available *mpt*64 Ct results, 60% of pulmonary TB cases and 75% of EPTB cases would have been anticipated to have successful amplicon MDR NGS testing. The remaining samples with no *mpt*64 Ct results available (secondary to initial testing in other clinical laboratories in BC) likely would have followed roughly the same distribution pattern.

The TATs for first-line phenotypic susceptibility testing (to isoniazid, rifampin, ethambutol, and moxifloxacin, all locally performed) were as described in Table 10, with roughly equivalent TAT for both AFB-Pos pulmonary TB and EPTB from sample collection date (29 and 30 days, respectively), but somewhat lower TAT for AFB-Pos EPTB cases relative to pulmonary TB cases from AFB smear result date, i.e., the date of processing in a laboratory (19 and 25 days, respectively).

The amplicon MDR NGS test at this time has not been implemented as a routine testing method on all samples eligible based on AFB smear/*mpt*64 Ct results and is performed on request only. Consequently, only three cases had direct amplicon MDR NGS testing performed at the time of data review. The mean TAT for MDR NGS results for these three samples was 11 days (about 1 and a half weeks) from sample collection date. The laboratory TAT of the test itself (which would have been the TAT for an AFB-Pos sample, if testing were to be implemented routinely) is 4 days from AFB smear result availability.

## 4. Discussion

Our amplicon-based NGS sequencing assay targeting MTB genes conferring resistance to antimicrobials of key clinical importance provides an excellent methodology for rapid determination of resistance/susceptibility predictions for AFB smear-positive cases. It demonstrated excellent performance with respect to all assessed parameters (sensitivity, specificity, accuracy, precision) and has been fully accredited by both the Diagnostic Accreditation Program (DAP—our jurisdiction’s regulatory body) and College of American Pathologists (CAP) for clinical testing. Identified discrepant results were primarily due to some mutations falling outside of the sequenced amplicons target area. This limitation, which is particularly notable for lack of coverage for *inh*A c.-154G > A mutation, associated with low level isoniazid resistance, can be addressed by further assay optimization, which is ongoing in our laboratory. Ultimate choice of targeted drugs to include in a laboratory developed amplicon tNGS assay would depend on local resistance epidemiology, access to additional testing modalities (e.g., WGS on growing isolates, phenotypic drug susceptibility testing) and jurisdictional treatment and isolation/de-isolation practices. Assay design that allows for ongoing optimization in response to changing needs is helpful to maintain high clinical utility.

Careful attention to quality parameters and good design of quality metrics can help identify potential artifacts, such as degenerate bases (which is a particularly important consideration in paucibacillary samples). Quality assurance-focused implementation with high confidence quality parameters helps mitigate against erroneous results reporting. However, as all sequencing outputs are accessible for review, detection of any mutations of concern that fall below the reportable threshold, still provides for an opportunity to discuss the findings with treating physicians and alert them to the possibility and to facilitate decision making with respect to ongoing samples collection and treatment regimens. Success rate of our amplicon tNGS assay was dependent on input material, with paucibacillary samples having a higher failure rate than high burden samples. This dependency is true to all diagnostic assays, and remains a diagnostic barrier, in particular for paucibacillary extrapulmonary TB cases. On the upside, successful sequencing of at least part of the amplicons with high predictive capacity for isoniazid and rifampin resistance was possible for 26/29 attempted primary samples, with 7 of these being extrapulmonary samples and 1 a gastric aspirate (Supplementary Table S5). Modifications to nucleic acid extraction protocols and template volumes can improve on LOD for diagnostic assays, including tNGS assays.

Cost effectiveness is an important consideration, especially in the current climate of economic uncertainty and budget cuts experienced in many jurisdictions. Our assay was designed to optimize price point and integration into the laboratory workflow, minimizing turnaround time by successful multiplexing of TB and non-tuberculous mycobacteria-focused targets. The current cost of our assay is about CAD 60, which is not much higher than the cost of GeneXpert^®^ cartridges commonly used for rapid MTB resistance predictions in North America, and about 10 times lower than our previously used Sanger sequencing-based assay. This cost-effectiveness makes our approach a very attractive option for implementation in reference laboratories that perform high volume of TB testing, which allows for optimal utilization of Illumina cartridges. Other laboratories have successfully implemented similar approaches on different sequencing platforms, such as Oxford Nanopore [16], which may be a more attractive approach for lower throughput testing. With ongoing technological optimization, further cost reductions are possible—e.g., modifications to library construction, such as Hackflex [27].

Our method not only provides information on specific mutations that are detected, but it has been adapted to also provide rapid identification of *M. bovis*/BCG strains (which has important implications for both clinical management, as well as infection control considerations) and provides higher accuracy for resistance assessments than typically used commercial molecular platforms. Depending on local MTB resistance epidemiology, resistance predictions from such platforms as GeneXpert^®^ and Truenat^®^ MTB have variable predictive values for actual MTB resistance. GeneXpert^®^ is known to pick up silent mutations in the rifampin resistance determining region and report them as “rifampin resistance” [28,29,30]. Similarly, we have recently experienced a case in our setting of a patient who was locally diagnosed with TB lymphadenitis, which was determined to be “Sensitive” to first-line drugs, however, was erroneously reported as “resistant” by a Truenat^®^ MTB test performed in a different jurisdiction. This patient had a c.-61C > T mutation detected upstream of the *rpo*B gene (not associated with rifampin resistance), which we suspect might have contributed to the erroneous Truenat^®^ MTB call; however, no resistance-conferring mutations were identified. With these considerations in mind, it becomes particularly salient for countries with low resistance burden to take particular care to confirm resistance predictions of commercial molecular systems using higher resolution technologies, such as NGS.

We have observed discrepancies between genotypic predictions and phenotypic susceptibility results for rifampin and ethambutol (Table 6), with detected mutations not translating into phenotypic resistance. Some mutations, in particular in *rpo*B gene, are known to not correspond to phenotypic resistance when tested using the currently CLSI-recommended critical concentrations of antimycobacterial drugs, with proposals from the WHO and tuberculosis practice groups for modification of phenotypic testing practices [31,32,33], although even modified critical concentrations do not always mitigate the genotypic to phenotypic correlation [34]. Future evaluations of genotypic–phenotypic correlations and clinically relevant breakpoints for *M. tuberculosis* resistance testing will help with future optimization of both tNGS and WGS assay outputs and their clinical predictive capacity.

The rapid TAT of our assay (4 days for the laboratory process, once the sample is available and is deemed eligible for assessment) provides a very attractive option for rapid and accurate determination of susceptibility/resistance patterns of smear-positive MTB disease. This proves very useful from the perspective of rapid initiation of appropriate treatment in resistant cases, as well as being able to facilitate rapid and timely discontinuation of isolation precautions for patients. The limitation of the assay is the high limit of detection that hinders resistance/susceptibility predictions for AFB smear-negative cases. This can be particularly limiting for EPTB disease, which is often paucibacillary [35]. However, there is still high utility for pulmonary TB, which is more often AFB smear-positive and results in onward transmission. In our setting, if this new targeted amplicon NGS method were to be routinely applied to all AFB smear-positive TB cases, we would expect on average 2.5–3 weeks faster TAT (for EPTB and pulmonary TB cases, respectively) for prediction of drug resistance/susceptibility. The ability to routinely implement this testing for all eligible cases, however, is dependent, among other things, on budgetary constraints.

Future work in this area should focus on further improvements to both the methodology’s performance characteristics (e.g., decreasing limit of detection to facilitate greater diagnostic applicability, expanding amplicon coverage for isoniazid resistance-conferring targets) and regularly assessing the local MTB susceptibility/resistance landscape for appropriate adaptation of selected gene targets to be of highest clinical utility. Our amplicon-based NGS pipeline can be easily adapted to incorporate and expand the list of selected genes and coverage of MTB mutations of increasing clinical importance.

## Figures and Tables

**Table 1:** Primer sequences and information. Rif. = rifampicin; Iso. = isoniazid; Pyr. = pyrazinamide; Eth. = ethambutol; Fluoro. = Fluoroquinolones including levofloxacin, moxifloxacin, ofloxacin, and ciprofloxacin.

Purpose	Target	Primer Name	Primer Sequence (5′-3′)	Final Conc. (μM)	Size (bp)	Refs.
Rif.	*rpo*B	*rpo*B-F	CTTGCACGAGGGTCAGACCA	0.1	503	[18]
		*rpo*B-R	ATCTCGTCGCTAACCACGCC	0.1		
Iso.	*kat*G	*kat*G-F	AACGACGTCGAAACAGCGGC	0.06	415	[18]
		*kat*G-R	GCGAACTCGTCGGCCAATTC	0.06		
	*inh*A/ *fab*G1	*inh*A P3.1	CTCCGGTAACCAGGACTGAACG	0.2	205	[19]
		*inh*A P5	CGCAGCCAGGGCCTCGCTG	0.2		
	*ndh*	*ndh*-F	ATCACCACCGCCGCTGAAGC	0.06	454	[20,21]
		*ndh*1AS	AATTCCGAGACGACGCACTG	0.06		
	*ahp*C	oxyR_*ahp*C-F_2009	GCCTGGGTGTTCGTCACTGGT	0.04	449	[20,22]
		oxyR_*ahp*C-R	GGTCGCGTAGGCAGTGCCCC	0.04		
Pyr.	*pnc*A	XDR_KA*pnc*A-F	GCGTCATGGACCCTATATCTGTGG	0.9	694	[23]
		XDR_KA*pnc*A-R	GTGAACAACCCGACCCAGC	0.9		
Eth.	*emb*B	XDR_*emb*B-3f	CTGACCGACGCCGTGGTGATAT	0.5	461	[18]
		XDR_*emb*B-4r	TGAATGCGGCGGTAACGACG	0.5		
Fluoro.	*gyr*A	XDR_*gyr*A-F	TCGACTATGCGATGAGCGTG	0.15	273	[24,25]
		XDR_NML_*gyr*A-2	GGGCTTCGGTGTACCTCAT	0.15		

**Table 2:** 2 × 2 contingency table for amplicon-based antimicrobial resistance calling through the tbProfiler pipeline. DR: drug resistance variants.

AMR by tbProfiler		WGS	
		DR	No DR
NGS	DR	42	2
	no DR	1	187

**Table 3:** Comparison of mutations detected by NGS and WGS, and drug resistance (DR) type called for each. Discrepants are highlighted in red. Mutations outside amplicon target regions are in grey. All variants had 100% of reads supporting the mutation.

Sample	DR Type		Discrepants	
	NGS	WGS	NGS	WGS
18s262	MDR-TB	MDR-TB		
18s423	MDR-TB	MDR-TB		
18s514	MDR-TB	MDR-TB		*inh* A p.I194T
19s078	RR-TB	RR-TB		
20s291	RR-TB	RR-TB		
20s304	MDR-TB	MDR-TB	*pnc* A p.P54S	
22s250	MDR-TB	MDR-TB		
22s291	Pre-XDR-TB	Pre-XDR-TB		
22s305	MDR-TB	MDR-TB		
22s317	Pre-XDR-TB	Pre-XDR-TB		*emb* B p.Q497R
22s320	RR-TB	RR-TB		
22s431	Pre-XDR-TB	Pre-XDR-TB		
23s071	MDR-TB	MDR-TB		
23s137	RR-TB	MDR-TB		*inh* A p.I194T
PS1	Sensitive	HR-TB		*inh* A c.-154G > A
PS3	Pre-XDR-TB	Pre-XDR-TB	*emb* B p.M306V	
PS4	Sensitive	HR-TB		*kat* G p.S315T
PS7	Sensitive	HR-TB		*inh* A c.-154G > A
PS9	Sensitive	HR-TB		*inh* A c.-154G > A
SPEC-34	Sensitive	Other		
SPEC-35	Sensitive	Sensitive		
SPEC-36	Sensitive	Sensitive		
SPEC-37	Sensitive	Sensitive		
SPEC-38	HR-TB	HR-TB		
SPEC-39	Sensitive	Sensitive		
SPEC-40	Sensitive	Sensitive		
SPEC-41	Sensitive	Sensitive		
SPEC-43	Sensitive	Sensitive		
SPEC-44	Sensitive	Sensitive		

**Table 4:** Summary of LOD and precision results for *M. tuberculosis* strain H37Rv (susceptible). Total replicates are indicated in brackets. Und = undetermined.

Dilution	Replicates Pass	Amplicons Detected	MPT64 Ct
10^−2^	3 (3)	8	24.33
10^−3^	3 (3)	8	27.83
10^−4^	9 (9)	8	31.29
10^−5^	9 (9)	8	35.81
10^−6^	2 (3)	3, 4	37.16
10^−7^	1 (3)	0	Und

**Table 5:** Comparison of NML susceptibility testing and tbProfiler results on NML isolates. MGIT = mycobacteria growth indicator tube method. Ethambutol tested at 5 μg/mL, pyrazinamide tested at 100 μg/mL, moxifloxacin (moxi.) tested at 0.25 μg/mL, and ofloxacin (oflo.) tested at 2 μg/mL. All variants had 100% (1.00) of reads supporting the mutation unless otherwise indicated in brackets.

Sample	Ethambutol		Pyrazinamide		Fluoroquinolones		
	NML	tbProfiler	NML	tbProfiler	NML (moxi.)	NML (oflo.)	tbProfiler
NML-XDR-1	Sensitive	-	Sensitive	-	Resistant	Resistant	*gyr*A p.D94Y
NML-XDR-2	Resistant	*emb*B p.G406D	Resistant	*pnc*A p.D49A (0.99)	Resistant	Resistant	*gyr*A p.D94H
NML-XDR-4	Sensitive	-	Resistant	*pnc*A p.D12A	Sensitive	Sensitive	-
NML-XDR-5	Sensitive	-	Resistant	*pnc*A p.H57D	Sensitive	Sensitive	-
NML-XDR-7	Sensitive	-	Resistant	*pnc*A p.H57D	Sensitive		-
NML-XDR-8	Sensitive	-	Resistant	*pnc*A p.V180G	Sensitive	Sensitive	-
NML-XDR-9	Sensitive	-	Sensitive	-	Sensitive	Sensitive	-
NML-XDR-10	Resistant	*emb*B p.M306V	Resistant	*pnc*A p.W119G	Sensitive	Sensitive	-
NML-XDR-11	Resistant	*emb*B p.M306I	Sensitive	-	Sensitive	Sensitive	-
NML-XDR-12	Sensitive	-	Resistant	*pnc*A p.V180G	Sensitive	Sensitive	-
NML-XDR-13	Sensitive	-	Sensitive	-	Resistant	Resistant	*gyr*A p.D94Y
NML-XDR-15	sensitive	-	Resistant	*pnc*A p.H57D	Sensitive		-

**Table 6:** Comparison of BCCDC susceptibility testing and tbProfiler results on validation samples. Red text indicates mismatch between BCCDC susceptibility and tbProfiler results. ND = not determined. All variants had 100% (1.00) of reads supporting the mutation unless otherwise indicated in brackets beside the variant.

Sample	Rifampicin		Isoniazid		Ethambutol		Pyrazinamide		Ofloxacin	
	MGIT 960	tbProfiler	MGIT 960	tbProfiler	MGIT 960	tbProfiler	MGIT 960	tbProfiler	MGIT 960	tbProfiler
18s262	R	*rpo*B p.S450L	R	*inh*A c.-777C > T	S	*emb* B p.G406D	S	-	S	-
18s423	R	*rpo*B p.S450; *rpo*B p.T400A	R	*inh*A c.-770T > C	S	-	R	-	S	-
18s514	S	*rpo* B p.L452P	R	*inh*A c.-777C > T	S	-	S	-	S	-
19s078	S	*rpo* B p.I480V	S	-	S	-	ND	-	S	-
20s291	S	*rpo* B p.H445Q	S	-	S	-	ND	-	S	-
20s304	R	*rpo*B p.S450L	R	*kat*G p.S315T	R	*emb*B p.M306V	ND	*pnc*A p.P54S	S	-
22s250	R	*rpo*B p.Q432K	R	*kat*G p.S315T	S	-	R	*pnc*A p.V139G	S	-
22s291	R	*rpo*B p.S450L	R	*kat*G p.S315T	R	*emb*B p.M306V	R	*pnc*A p.L182S	R	*gyr*A p.A90V (0.99)
22s305	R	*rpo*B p.S450L	R	*inh*A c.-777C > T	S	*emb* B p.M306I	S	-	S	-
22s317	R	*rpo*B p.S450L	R	*inh*A c.-777C > T; *kat*G p.S315T	R	-	R	*pnc*A p.G132A	R	*gyr*A p.D94G
22s320	R	*rpo*B p.D435V	S	-	S	-	S	-	S	-
22s431	R	*rpo*B p.S450L	R	*kat*G p.S315T	R	*emb*B p.M306V	R	*pnc*A p.D63G	R	*gyr*A p.D94A
23s071	R	*rpo*B p.S450L	R	*kat*G p.S315T	S	-	S	-	S	-
23s137	R	*rpo*B p.Q432P	R	-	S	-	S	-	S	-
PS1	S	-	R	-	S	-	S	-	S	-
PS3	R	*rpo*B p.S450L	R	*kat*G p.S315T	R	*emb*B p.M306V	R	*pnc*A p.L182S	R	*gyr*A p.A90V
PS4	S	-	R	-	S	-	ND	-	S	-
PS7	S	-	R	-	S	-	ND	-	S	-
PS9	S	-	R	-	S	-	ND	-	S	-
SPEC-34	S	-	S	-	S	-	ND	-	S	-
SPEC-35	S	-	S	-	S	-	ND	-	S	-
SPEC-36	S	-	S	-	S	-	ND	-	S	-
SPEC-37	S	-	S	-	S	-	ND	-	S	-
SPEC-38	S	-	R	*inh*A c.-777C > T	S	-	S	-	S	-
SPEC-39	S	-	S	-	S	-	ND	-	S	-
SPEC-40	S	-	S	-	S	-	ND	-	S	-
SPEC-41	S	-	S	-	S	-	ND	-	S	-
SPEC-43	S	-	S	-	S	-	ND	-	S	-
SPEC-44	S	-	S	-	S	-	ND	-	S	-

**Table 7:** Amplicon results and drug resistant (DR) type predicted for post-implementation samples. Low confidence DR types (due to <8 amplicons sequenced) are in grey. All samples had 100% median coverage of amplicons sequenced and DR variants were (1.00). REV/P = REVIEW/REPEAT; AST = antimicrobial susceptibility testing; Rif = rifampicin; Iso = isoniazid; Moxi = moxifloxacin; Oflo = ofloxacin; Eth = ethambutol; Pyr = pyrazinamide. Phenotypic AST not completed for this sample. Green background highlights samples that passed QC of tNGS testing and red background highlights samples that were flagged for review and repeat.

Sample	NGS	Amplicons	DR Type	DR Variants	Phenotypic AST
Direct					
CS_1	PASS	8	Sensitive		Sensitive
CS_2	PASS	8	Sensitive		Sensitive
CS_3	PASS	8	HR-TB	*inh*A c.-777C > T	-
CS_5	PASS	8	Sensitive		Sensitive
CS_8	PASS	8	Sensitive		Sensitive
CS_9	PASS	8	MDR-TB	*rpo*B p.S450L *kat*G p.S315T	Rif-, Iso-, Eth-resistant
CS_23	PASS	8	Sensitive		Moxi-resistant
CS_24	PASS	8	Sensitive		Sensitive
CS_25	PASS	8	Sensitive		Sensitive
CS_26	PASS	8	Sensitive		Sensitive
CS_27	PASS	8	Other	*gyr*A p.Ala90Val	Oflo-resistant
CS_28	PASS	8	Sensitive		Sensitive
CS_29	PASS	8	Sensitive		-
CS_30	PASS	8	Sensitive		Sensitive
CS_31	PASS	8	Sensitive		Sensitive
CS_35	PASS	8	Other	*pnc*A p.His57Asp	Pyr-resistant
CS_36	PASS	8	Sensitive		Sensitive
CS_37	PASS	8	Sensitive		-
CS_38	PASS	8	Sensitive		Sensitive
CS_41	PASS	8	Other	*pnc*A p.His57Asp	Pyr-resistant
CS_43	PASS	8	Other	*pnc*A p.His57Asp	-
CS_4	REV/P	7	Sensitive		Sensitive
CS_6	REV/P	5	Sensitive		Sensitive
CS_7	REV/P	7	RR-TB	*rpo*B p.S450L	Rif-resistant
CS_32	REV/P	7	Other	*pnc*A p.His57Asp	Pyr-resistant
CS_33	REV/P	5	RR-TB	*rpo*B p.Ser450Leu	-
CS_34	REV/P	7	Sensitive		-
CS_40	REV/P	3	Sensitive		-
CS_42	REV/P	2	Sensitive		-
Culture					
23H1076	PASS	8	Sensitive		Sensitive
23H1106	PASS	8	Sensitive		Sensitive
23H1107	PASS	8	Other	*pnc*A p.H57D	Pyr-resistant
23H1169	PASS	8	Sensitive		Sensitive
23H911	PASS	8	Sensitive		Sensitive
23H974	PASS	8	Sensitive		Sensitive
23H915	REV/P	7	Sensitive		-
24H61	REV/P	7	Other	*pnc* A p.H57D	Pyr-resistant

**Table 8:** pncA p.His57Asp mutation calling for assignment of *M. bovis* BCG genotype. Here, presumptive BCG refers to ID of the sample as “presumptive *M. bovis*/*M. bovis* BCG”.

Sample	Mutation	NGS	Reference
Validation samples			
NML-XDR-15	p.His57Asp	Presumptive BCG	Mycobacterium_bovis_BC
NML-XDR-5	p.His57Asp	Presumptive BCG	Mycobacterium_bovis_BC
NML-XDR-7	p.His57Asp	Presumptive BCG	Mycobacterium_bovis_BC
Post-implementation samples			
23H1107	p.His57Asp	Presumptive BCG	
24H61	p.His57Asp	Presumptive BCG	
CS_35	p.His57Asp	Presumptive BCG	
CS_41	p.His57Asp	Presumptive BCG	
CS_43	p.His57Asp	Presumptive BCG	
CS_32	p.His57Asp	Presumptive BCG	

**Table 9:** Acid fast bacilli and TB PCR results characteristics of patients included for review.

	Pulmonary TB *n* (% Total)	EPTB *n* (% Total)
AFB-Pos and *mpt*64 ≤ 33	42 (33.6%)	9 (40.9%)
AFB-Pos and *mpt*64 > 33	28 (22.4%)	3 (13.6%)
AFB-Pos and *mpt*64 Ct results N/A	55 (44%)	10 (45%)

**Table 10:** Turnaround times for first line antibiotics susceptibility results of pulmonary TB and EPTB cases.

	Pulmonary TB		EPTB	
	AFB-Pos	All Cases	AFB-Pos	All Cases
TAT from sample collection (days)	29	34	30	40
TAT from AFB smear results (days)	25	29	19	29

## Data Availability

Data is provided as part of this manuscript/Supplementary Materials; if any further information is required, please contact the corresponding author.

## Supplementary Materials

The following supporting information can be downloaded at: https://www.mdpi.com/article/10.3390/tropicalmed10070194/s1, Figure S1: Alignment of reads with the low confidence SNP in *pnc*A; Table S1: *Mycobacterium tuberculosis* complex validation samples; Table S2: All drug-resistant mutations detected by NGS in validation sample set shown by amplicon; Table S3: All drug-resistant mutations detected by WGS in validation sample set shown by amplicon relevant to NGS data (i.e., omitted drug-resistant mutations detected in genes not included in NGS assay); Table S4: H37Rv strain (sensitive) AMR amplicon and mutation results; Table S5: Post-implementation clinical M. tuberculosis complex (MTBC) samples.

## References

[1] Barberis I., Bragazzi N.L., Galluzzo L., Martini M. (2017). The History of Tuberculosis: From the First Historical Records to the Isolation of Koch’s Bacillus. J. Prev. Med. Hyg..

[2] Implementing the End TB Strategy: The Essentials, 2022 Update. https://www.who.int/publications/i/item/9789240065093.

[3] The Untold Story: New Report Reveals 7000 Additional TB Deaths During COVID-19 Pandemic. https://www.who.int/europe/news/item/21-03-2024-the-untold-story--new-report-reveals-7000-additional-tb-deaths-during-covid-19-pandemic.

[4] World Health Organisation (2020). Global Tuberculosis Report 2020.

[5] Walker T.M., Miotto P., Köser C.U., Fowler P.W., Knaggs J., Iqbal Z., Hunt M., Chindelevitch L., Farhat M., Cirillo D.M. (2022). The 2021 WHO Catalogue of *Mycobacterium Tuberculosis* Complex Mutations Associated with Drug Resistance: A Genotypic Analysis. Lancet Microbe.

[6] World Health Organization (2023). Catalogue of Mutations in Mycobacterium Tuberculosis Complex and Their Association with Drug Resistance.

[7] Cooper R. (2022). Appendix B: De-Isolation Review and Recommendations. Can. J. Respir. Crit. Care Sleep Med..

[8] Shah M., Dansky Z., Nathavitharana R., Behm H., Brown S., Dov L., Fortune D., Gadon N.L., Gardner Toren K., Graves S. (2024). NTCA Guidelines for Respiratory Isolation and Restrictions to Reduce Transmission of Pulmonary Tuberculosis in Community Settings. Clin. Infect. Dis..

[9] WHO (2024). Consolidated Guidelines on Tuberculosis: Module 3: Diagnosis: Rapid Diagnostics for Tuberculosis Detection.

[10] Target Product Profile for Next-Generation Drug-Susceptibility Testing at Peripheral Centres. https://www.who.int/publications/i/item/9789240032361.

[11] Schwab T.C., Perrig L., Göller P.C., De la Hoz F.F.G., Lahousse A.P., Minder B., Günther G., Efthimiou O., Omar S.V., Egger M. (2024). Targeted Next-Generation Sequencing to Diagnose Drug-Resistant Tuberculosis: A Systematic Review and Meta-Analysis. Lancet Infect. Dis..

[12] Gliddon H.D., Frampton D., Munsamy V., Heaney J., Pataillot-Meakin T., Nastouli E., Pym A.S., Steyn A.J.C., Pillay D., McKendry R.A. (2021). A Rapid Drug Resistance Genotyping Workflow for *Mycobacterium tuberculosis*, Using Targeted Isothermal Amplification and Nanopore Sequencing. Microbiol. Spectr..

[13] Song J., Du W., Liu Z., Che J., Li K., Che N. (2022). Application of Amplicon-Based Targeted NGS Technology for Diagnosis of Drug-Resistant Tuberculosis Using FFPE Specimens. Microbiol. Spectr..

[14] Barbosa-Amezcua M., Cuevas-Córdoba B., Fresno C., Haase-Hernández J.I., Carrillo-Sánchez K., Mata-Rocha M., Muñoz-Torrico M., Bäcker C., González-Covarrubias V., Alaez-Verson C. (2022). Rapid Identification of Drug Resistance and Phylogeny in M. Tuberculosis, Directly from Sputum Samples. Microbiol. Spectr..

[15] Leung K.S.-S., Tam K.K.-G., Ng T.T.-L., Lao H.-Y., Shek R.C.-M., Ma O.C.K., Yu S.-H., Chen J.-X., Han Q., Siu G.K.-H. (2022). Clinical Utility of Target Amplicon Sequencing Test for Rapid Diagnosis of Drug-Resistant *Mycobacterium tuberculosis* from Respiratory Specimens. Front. Microbiol..

[16] Murphy S.G., Smith C., Lapierre P., Shea J., Patel K., Halse T.A., Dickinson M., Escuyer V., Rowlinson M.C., Musser K.A. (2023). Direct Detection of Drug-Resistant *Mycobacterium tuberculosis* Using Targeted next Generation Sequencing. Front. Public Health.

[17] Canada, P.H.A. of Antimicrobial Resistance: Seasonal Update—Canada.ca. https://health-infobase.canada.ca/carss/amr/results.html?ind=11.

[18] Lu W., Feng Y., Wang J., Zhu L. (2016). Evaluation of *MTBDRplus* and *MTBDRsl* in Detecting Drug-Resistant Tuberculosis in a Chinese Population. Dis. Markers.

[19] Oudghiri A., Karimi H., Chetioui F., Zakham F., Bourkadi J.E., Elmessaoudi M.D., Laglaoui A., Chaoui I., El Mzibri M. (2018). Molecular Characterization of Mutations Associated with Resistance to Second-Line Tuberculosis Drug among Multidrug-Resistant Tuberculosis Patients from High Prevalence Tuberculosis City in Morocco. BMC Infect. Dis..

[20] Bakhtiyariniya P., Khosravi A.D., Hashemzadeh M., Savari M. (2022). Detection and Characterization of Mutations in Genes Related to Isoniazid Resistance in *Mycobacterium tuberculosis* Clinical Isolates from Iran. Mol. Biol. Rep..

[21] Lee A.S., Teo A.S., Wong S.Y. (2001). Novel Mutations in *ndh* in Isoniazid-Resistant *Mycobacterium tuberculosis* Isolates. Antimicrob. Agents Chemother..

[22] Valvatne H., Syre H., Kross M., Stavrum R., Ti T., Phyu S., Grewal H.M.S. (2009). Isoniazid and Rifampicin Resistance-Associated Mutations in *Mycobacterium tuberculosis* Isolates from Yangon, Myanmar: Implications for Rapid Molecular Testing. J. Antimicrob. Chemother..

[23] Streicher E.M., Maharaj K., York T., Van Heerden C., Barnard M., Diacon A., Mendel C.M., Bosman M.E., Hepple J.A., Pym A.S. (2014). Rapid Sequencing of the *Mycobacterium tuberculosis pnc*A Gene for Detection of Pyrazinamide Susceptibility. J. Clin. Microbiol..

[24] Cheng A.F.B., Yew W.W., Chan E.W.C., Chin M.L., Hui M.M.M., Chan R.C.Y. (2004). Multiplex PCR Amplimer Conformation Analysis for Rapid Detection of *gyr*A Mutations in Fluoroquinolone-Resistant *Mycobacterium tuberculosis* Clinical Isolates. Antimicrob. Agents Chemother..

[25] Hu Y., Xu L., He Y.L., Pang Y., Lu N., Liu J., Shen J., Zhu D.M., Feng X., Wang Y.W. (2017). Prevalence and Molecular Characterization of Second-Line Drugs Resistance among Multidrug-Resistant *Mycobacterium tuberculosis* Isolates in Southwest of China. BioMed Res. Int..

[26] EP12|Evaluation of Qualitative, Binary Output Examination Performance. https://clsi.org/shop/standards/ep12/.

[27] Gaio D., Anantanawat K., To J., Liu M., Monahan L., Darling A.E. (2022). Hackflex: Low-Cost, High-Throughput, Illumina Nextera Flex Library Construction. Microb. Genom..

[28] Kense A., Jorgensen D., Hird T., KhunKhun R., Lam M., Kibsey P., Rodrigues M., Sekirov I. (2024). Discordance in rifampin resistance determination between a commercial molecular test, whole genome sequencing, and phenotypic testing, for a provincially circulating *Mycobacterium tuberculosis* strain. J. Assoc. Med. Microbiol. Infect. Dis. Can..

[29] Mathys V., van de Vyvere M., de Droogh E., Soetaert K., Groenen G. (2014). False-Positive Rifampicin Resistance on Xpert® MTB/RIF Caused by a Silent Mutation in the *rpo*B Gene. Int. J. Tuberc. Lung Dis..

[30] Mokaddas E., Ahmad S., Eldeen H.S., Al-Mutairi N. (2015). Discordance between Xpert MTB/RIF Assay and Bactec MGIT 960 Culture System for Detection of Rifampin-Resistant *Mycobacterium tuberculosis* Isolates in a Country with a Low Tuberculosis (TB) Incidence. J. Clin. Microbiol..

[31] WHO Announces Updated Critical Concentrations for Susceptibility Testing to Rifampicin. https://www.who.int/news/item/05-02-2021-who-announces-updated-critical-concentrations-for-susceptibility-testing-to-rifampicin.

[32] Technical Report on Critical Concentrations for Drug Susceptibility Testing of Medicines Used in the Treatment of Drug-Resistant Tuberculosis. https://www.who.int/publications/i/item/WHO-CDS-TB-2018.5.

[33] Antimycobacterial Susceptibility Testing Group (2022). Updating the Approaches to Define Susceptibility and Resistance to Anti-Tuberculosis Agents: Implications for Diagnosis and Treatment. Eur. Respir. J..

[34] Rupasinghe P., Ashraf A., Barreda N., Parveen S., Zubair M., Calderon R., Asif S., Hirani N., Chingisova L., Bulane A. (2024). Reduced Critical Concentration Might Not Have Improved MGIT-Based DST’s Sensitivity to Rifampicin. Antimicrob. Agents Chemother..

[35] Jain R., Gupta G., Mitra D.K., Guleria R. (2024). Diagnosis of Extra Pulmonary Tuberculosis: An Update on Novel Diagnostic Approaches. Respir. Med..

