# Supplementary material for: Series 2: Development of a Multiplex Amplicon Next Generation Sequencing Assay for Rapid Assessment of Resistance-Associated Mutations in M. tuberculosis Clinical Cases
Source: Trop Med Infect Dis. 2025 Jul 10;10(7):194. doi: 10.3390/tropicalmed10070194 (PMC12300215; doi:10.3390/tropicalmed10070194)
Supplement: Supplementary file 1 [file tropicalmed-10-00194-s001.zip › tropicalmed-3647569-supplementary.pdf]

Supplementary Table S1. *Mycobacterium tuberculosis* complex validation samples.

Sample	NGS	Amplicons	Median coverage	Median depth	Type
18s262	PASS	8	100.0	4590	Culture
18s423	PASS	8	100.0	2101	Culture
18s514	PASS	8	100.0	3673	Culture
19s078	PASS	8	100.0	1762	Culture
20s291	PASS	8	100.0	3986	Culture
20s304	PASS	8	100.0	3500	Culture
22s250	PASS	8	100.0	6136	Culture
22s291	PASS	8	100.0	3838	Culture
22s305	PASS	8	100.0	5452	Culture
22s317	PASS	8	100.0	6587	Culture
22s320	PASS	8	100.0	6944	Culture
22s431	PASS	8	100.0	5516	Culture
23s071	PASS	8	100.0	3957	Culture
23s137	PASS	8	100.0	6581	Culture
NML-XDR-1	PASS	8	100.0	2245	NML isolate
NML-XDR-2	PASS	8	100.0	1047	NML isolate
NML-XDR-4	PASS	8	100.0	1041	NML isolate
NML-XDR-5	PASS	8	100.0	3807	NML isolate
NML-XDR-6	REVIEW/REPEAT	3	100.0	5656	NML isolate
NML-XDR-7	PASS	8	100.0	3695	NML isolate
NML-XDR-8	PASS	8	100.0	3363	NML isolate
NML-XDR-9	PASS	8	100.0	2219	NML isolate
NML-XDR-10	PASS	8	100.0	2152	NML isolate
NML-XDR-11	PASS	8	100.0	2382	NML isolate
NML-XDR-12	PASS	8	100.0	2162	NML isolate
NML-XDR-13	PASS	8	100.0	2275	NML isolate
NML-XDR-14	PASS	8	100.0	2205	NML isolate

NML-XDR-15	PASS	8	100.0	2157	NML isolate
NML-XDR-16	PASS	8	100.0	2686	NML isolate
NML-XDR-17	PASS	8	100.0	2332	NML isolate
PS1	PASS	8	100.0	1396	Direct (ascitic fluid)
PS3	PASS	8	100.0	507	Direct (sputum)
PS4	PASS	8	100.0	3402	Direct (sputum)
PS7	PASS	8	100.0	2222	Direct (sputum)
PS8	REVIEW/REPEAT	7	100.0	587	Direct (neck abscess)
PS9	PASS	8	100.0	1967	Direct (sputum)
PS10	REVIEW/REPEAT	7	98.4	5	Direct (sputum)
SPEC-34	PASS	8	100.0	758	Direct (sputum)
SPEC-35	PASS	8	100.0	1668	Direct (bronchial washings)
SPEC-36	PASS	8	100.0	201	Direct (sputum)
SPEC-37	PASS	8	100.0	1023	Direct (cecum tissue)
SPEC-38	PASS	8	100.0	799	Direct (pleural fluid)
SPEC-39	PASS	8	100.0	1200	Direct (bronchial washings)
SPEC-40	PASS	8	100.0	1370	Direct (sputum)
SPEC-41	PASS	8	100.0	1140	Direct (sputum)
SPEC-43	PASS	8	100.0	16	Direct (sputum)
SPEC-44	PASS	8	100.0	125	Direct (sputum)

Supplementary Table S2. All drug-resistant mutations detected by NGS in validation sample set shown by amplicon. Samples that do not have a reference (through WGS) are shaded in grey. All variants had 100% (1.00) of reads supporting the mutation unless otherwise indicated in brackets.

**Sample**	***rpoB***	***katG***	***inha/fabG1***	***ndh***	***ahpC***	***pncA***	***embB***	***gyrA***
18s262	rpoB p.S450L	-	inhA c.777C>T	-	-	-	embB p.G406D	-

18s423	rpoB p.S450W; rpoB p.T400A	-	inhA c.770T>C	-	-	-	-	-
18s514	rpoB p.L452P	-	inhA c.777C>T	-	-	-	-	-
19s078	rpoB p.I480V	-	-	-	-	-	-	-
20s291	rpoB p.H445Q	-	-	-	-	-	-	-
20s304	rpoB p.S450L	katG p.S315T	-	-	-	pncA p.P54S	embB p.M306V	-
22s250	rpoB p.Q432K	katG p.S315T	-	-	-	pncA p.V139G	-	-
22s291	rpoB p.S450L	katG p.S315T	-	-	-	pncA p.L182S	embB p.M306V	gyrA p.A90V (0.99)
22s305	rpoB p.S450L	-	inhA c.777C>T	-	-	-	embB p.M306I	-
22s317	rpoB p.S450L	katG p.S315T	inhA c.777C>T	-	-	pncA p.G132A	-	gyrA p.D94G
22s320	rpoB p.D435V	-	-	-	-	-	-	-
22s431	rpoB p.S450L	katG p.S315T	-	-	-	pncA p.D63G	embB p.M306V	gyrA p.D94A
23s071	rpoB p.S450L	katG p.S315T	-	-	-	-	-	-
23s137	rpoB p.Q432P	-	-	-	-	-	-	-
NML-XDR-1	-	-	inhA c.777C>T	-	-	-	-	gyrA p.D94Y
NML-XDR-2	-	katG p.S315T; katG p.T275A	-	-	-	pncA p.D49A (0.99)	embB p.G406D	gyrA p.D94H
NML-XDR-4	-	katG p.S315T	-	-	-	pncA p.D12A	-	-
NML-XDR-5	-	-	-	-	-	pncA p.H57D	-	-
NML-XDR-7	-	-	-	-	-	pncA p.H57D	-	-
NML-XDR-8	-	-	-	-	-	pncA p.V180G	-	-
NML-XDR-9	-	katG p.S315T	-	-	-	-	-	-
NML-XDR-10	rpoB p.S450W	katG p.S315T	inhA c.770T>C	-	-	pncA p.W119G	embB p.M306V	-
NML-XDR-11	-	katG p.S315T	-	-	ahpC c.48G>A	-	embB p.M306I	-
NML-XDR-12	-	-	-	-	-	pncA p.V180G	-	-
NML-XDR-13	-	-	-	-	-	-	-	gyrA p.D94Y
NML-XDR-14	rpoB p.S450L	katG p.S315T	-	-	-	pncA p.V128G	embB p.M306V	-
NML-XDR-15	-	-	-	-	-	pncA p.H57D	-	-
NML-XDR-16	-	-	-	-	-	-	-	-
NML-XDR-17	-	-	-	-	-	-	embB p.M306V	-
PS1	-	-	-	-	-	-	-	-
PS3	rpoB p.S450L	katG p.S315T	-	-	-	pncA p.L182S	embB p.M306V	gyrA p.A90V
PS4	-	-	-	-	-	-	-	-
PS7	-	-	-	-	-	-	-	-
PS9	-	-	-	-	-	-	-	-
SPEC-34	-	-	-	-	-	-	-	-
SPEC-35	-	-	-	-	-	-	-	-

SPEC-36	-	-	-	-	-	-	-	-	-
SPEC-37	-	-	-	-	-	-	-	-	-
SPEC-38	-	-	inhA c.777C>T	-	-	-	-	-	-
SPEC-39	-	-	-	-	-	-	-	-	-
SPEC-40	-	-	-	-	-	-	-	-	-
SPEC-41	-	-	-	-	-	-	-	-	-
SPEC-43	-	-	-	-	-	-	-	-	-
SPEC-44	-	-	-	-	-	-	-	-	-

Supplementary Table S3. All drug resistant mutations detected by WGS in validation sample set shown by amplicon relevant to NGS data (i.e. omitted drug-resistant mutations detected in genes not included in NGS assay). Note that fabG1 c.-8T>C is an alias for inhA c.770T>C and fabG1 c.-15C>T is an alias for inhA c.777C>T. All variants had 100% (1.00) of reads supporting the mutation unless otherwise indicated in brackets.

**sample**	***rpoB***	***katG***	***inha/fabG1***	***ndh***	***ahpC***	***pncA***	***embB***	***gyrA***
18s262	rpoB p.S450L	-	fabG1 c.-15C>T	-	-	-	embB p.G406D	-
18s423	rpoB p.T400A; rpoB p.S450W (0.99)	-	fabG1 c.-8T>C	-	-	-	-	-
18s514	rpoB p.L452P	-	fabG1 c.-15C>T; inhA p.I194T	-	-	-	-	-
19s078	rpoB p.I480V	-	-	-	-	-	-	-
20s291	rpoB p.H445Q	-	-	-	-	-	-	-
20s304	rpoB p.S450L	katG p.S315T	-	-	-	-	embB p.M306V	-
22s250	rpoB p.Q432K	katG p.S315T	-	-	-	pncA p.V139G	-	-
22s291	rpoB p.S450L	katG p.S315T	-	-	-	pncA p.L182S	embB p.M306V	gyrA p.A90V
22s305	rpoB p.S450L	-	fabG1 c.-15C>T	-	-	-	embB p.M306I	-
22s317	rpoB p.S450L	katG p.S315T	fabG1 c.-15C>T	-	-	pncA p.G132A	embB p.Q497R	gyrA p.D94G
22s320	rpoB p.D435V (0.99)	-	-	-	-	-	-	-
22s431	rpoB p.S450L	katG p.S315T	-	-	-	pncA p.D63G	embB p.M306V	gyrA p.D94A
23s071	rpoB p.S450L	katG p.S315T	-	-	-	-	-	-
23s137	rpoB p.Q432P (0.99)	-	inhA p.I194T	-	-	-	-	-
PS1	-	-	inhA c.-154G>A	-	-	-	-	-

PS3	rpoB p.S450L	katG p.S315T	-	-	-	pncA p.L182S	-	gyrA p.A90V
PS4	-	katG p.S315T (0.67)	-	-	-	-	-	-
PS7	-	-	inhA c.-154G>A	-	-	-	-	-
PS9	-	-	inhA c.-154G>A	-	-	-	-	-
SPEC-34	-	-	-	-	-	-	-	-
SPEC-35	-	-	-	-	-	-	-	-
SPEC-36	-	-	-	-	-	-	-	-
SPEC-37	-	-	-	-	-	-	-	-
SPEC-38	-	-	fabG1 c.-15C>T	-	-	-	-	-
SPEC-39	-	-	-	-	-	-	-	-
SPEC-40	-	-	-	-	-	-	-	-
SPEC-41	-	-	-	-	-	-	-	-
SPEC-43	-	-	-	-	-	-	-	-
SPEC-44	-	-	-	-	-	-	-	-

Supplementary Table S4. H37Rv strain (sensitive) AMR amplicon and mutation results. All amplicons had a median coverage of 100%. Proportion of reads supporting mutations is in brackets in drug resistant (DR) Variants column. NGS refers to QC metrics for amplicon sequencing.

Sample	Dilution	Replicate	NGS	Amplicons	Median depth	DR type	Percent mapped reads	DR Variants
H37Rv-2-a	2	a	PASS	8	3527	Sensitive	98.7	
H37Rv-2-b	2	b	PASS	8	2986	Sensitive	98.6	
H37Rv-2-c	2	c	PASS	8	3136	Sensitive	99.2	
H37Rv-3-a	3	a	PASS	8	4154	Sensitive	99.1	
H37Rv-3-b	3	b	PASS	8	4317	Sensitive	99.2	
H37Rv-3-c	3	c	PASS	8	3896	Sensitive	99.0	
H37Rv-4-a	4	a	PASS	8	19757	Sensitive	98.6	
H37Rv-4-b	4	b	PASS	8	4683	Sensitive	98.6	
H37Rv-4-c	4	c	PASS	8	5039	Sensitive	98.9	
H37Rv-5-a	5	a	PASS	8	4567	Sensitive	98.9	

H37Rv-5-b	5	b	PASS	8	4452	Sensitive	98.3	
H37Rv-5-c	5	c	PASS	8	3750	Sensitive	98.8	
H37Rv-6-b	6	b	REVIEW/REPEAT	3	8466	Sensitive	95.9	
H37Rv-6-c	6	c	REVIEW/REPEAT	4	7891	Sensitive	97.1	
H37Rv-7-a	7	a	REVIEW/REPEAT	0		Sensitive	75.1	
H37-4-a	4	a	PASS	8	1706	Sensitive	98.9	
H37-4-b	4	b	PASS	8	1131	Sensitive	98.9	
H37-4-c	4	c	PASS	8	1677	Sensitive	99.1	
H37-5-a	5	a	PASS	8	1629	Sensitive	97.1	
H37-5-b	5	b	PASS	8	1214	Other	98.4	pncA p.D136Y (0.53)
H37-5-c	5	c	PASS	8	1129	Sensitive	98.9	
P2-H37-4-a	4	a	PASS	8	3381	Sensitive	99.2	
P2-H37-4-b	4	b	PASS	8	3059	Sensitive	98.6	
P2-H37-4-c	4	c	PASS	8	3584	Sensitive	99.0	
P2-H37-5-a	5	a	PASS	8	1618	Sensitive	98.8	
P2-H37-5-b	5	b	PASS	8	1530	Sensitive	99.3	
P2-H37-5-c	5	c	PASS	8	1394	Sensitive	98.7	

Supplementary Table S5. Post-implementation clinical *M. tuberculosis* complex (MTBC) samples. MTBC was also confirmed through *hsp65* speciation sequencing. Scroll = formalin-fixed paraffin-embedded tissue cut as scroll, CAP = proficiency sample designed to mimic sputum, BW = bronchiolar wash, UTUC = urogenital tract urothelial carcinoma, BAL = bronchoalveolar lavage. TNP = test not performed. AST = antimicrobial susceptibility testing. Rif = rifampicin, INH = isoniazide, EMB = ethambutol, NA = not applicable.

Sample ID	NGS	Failed amplicons	Median depth	Percent mapped reads	Sample Type	Smear result	MPT64 Ct	Culture result

CS_1	PASS		6307	99.8	Tissue	Neg	TNP	TB
CS_2	PASS		6500	99.7	Sputum	4+	NA	TB
CS_3	PASS		6153	99.8	Scroll	NA	24.28	No culture setup
CS_4	REVIEW/REPEAT	gyrA	455	37	Gastric Aspirate	1+	35.58	TB
CS_5	PASS		3861	92.2	Sputum	4+	NA	TB
CS_6	REVIEW/REPEAT	gyrA, pncA, rpoB	3843	98.3	CAP SAMPLE	NA	33.78	TB
CS_7	REVIEW/REPEAT	gyrA	3639	99.6	CAP SAMPLE	NA	34.61	TB
CS_8	PASS		1772	99.7	BW	1+	NA	TB
CS_9	PASS		197	30.5	Sputum	1+	34.85	TB
CS_23	PASS		6093		Sputum	4+	20.82	
CS_24	PASS		6685		Sputum	4+	22.90	
CS_25	PASS		13840		Sputum	4+	23.48	
CS_26	PASS		5314		BW	4+	26.01	
CS_27	PASS		3825		Tissue	3+	27.50	
CS_28	PASS		1578		Sputum	4+	28.23	
CS_29	PASS		374		Sputum	3+	29.62	
CS_30	PASS		459		Abscess	2+	31.42	
CS_31	PASS		4239		Sputum	NA	31.83	
CS_32	REVIEW/REPEAT	gyrA	251		Sputum	2+	32.35	
CS_33	REVIEW/REPEAT	embB, gyrA, ahpc	3153		Sputum	NA	33.30	
CS_34	REVIEW/REPEAT	gyrA	6000		Sputum	NA	33.32	
CS_35	PASS		7338		UTUC	1+(>10)	33.38	
CS_36	PASS		213		Sputum	NA	33.53	
CS_37	PASS		12		Sputum	1+(>10)	33.89	
CS_38	PASS		344		BAL	2+	34.46	
CS_40	REVIEW/REPEAT	pncA, katG, ndh, ahpc, rpoB	486		Sputum	NA	36.10	
CS_41	PASS		4699		Tissue	1+(<10)	Und	

CS_42	REVIEW/REPEAT	gyrA, pncA, katG, ndh, ahpc, rpoB	1227		Sputum	N/A	Und	
CS_43	PASS		2051		Scroll	N/A	Und	
23H1076	PASS		9947	99.8	Culture			TB
23H1106	PASS		5909	99.7	Culture			TB
23H1107	PASS		4307	99.8	Culture			BCG
23H1169	PASS		5455	97.9	Culture			TB
23H911	PASS		8846	99.8	Culture			TB
23H915	REVIEW/REPEAT	gyrA	91	99.6	Culture			TB
23H974	PASS		10334	99.7	Culture			TB
24H61	REVIEW/REPEAT	gyrA	6556	99.8	Culture			BCG

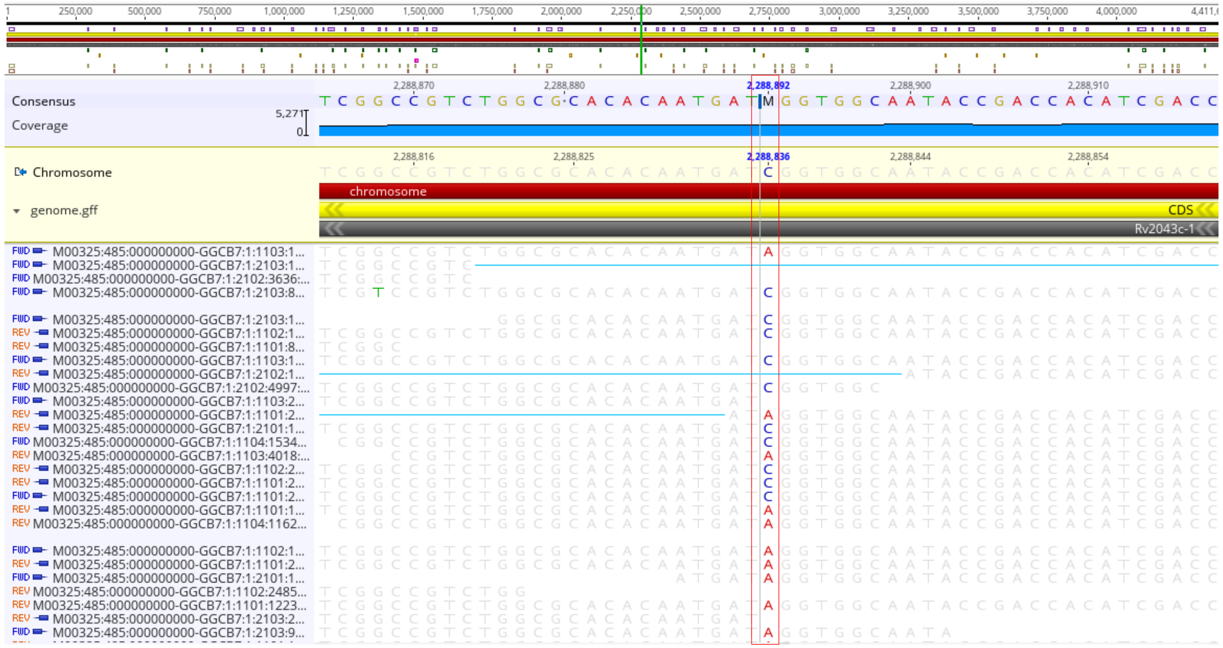

Supplementary Figure S1. Alignment of reads with the low confidence SNP in pncA.
